# Short-Wave Near-Infrared Spectrometer for Alcohol Determination and Temperature Correction

**DOI:** 10.1155/2012/728128

**Published:** 2012-05-08

**Authors:** Qingbo Fu, Jinming Wang, Guannan Lin, Hui Suo, Chun Zhao

**Affiliations:** State Key Laboratory on Integrated Optoelectronics, College of Electronic Science and Engineering, Jilin University, 2699 Qianjin Street, Changchun 130012, China

## Abstract

A multichannel short-wave near-infrared (SW-NIR) spectrometer module based on charge-coupled device (CCD) detection was designed. The design relied on a tungsten lamp enhanced by light emitting diodes, a fixed grating monochromator and a linear CCD array. The main advantages were high optical resolution and an optimized signal-to-noise ratio (0.24 nm and 500, resp.) in the whole wavelength range of 650 to 1100 nm. An application to alcohol determination using partial least squares calibration and the temperature correction was presented. It was found that the direct transfer method had significant systematic prediction errors due to temperature effect. Generalized least squares weighting (GLSW) method was utilized for temperature correction. After recalibration, the RMSEP found for the 25°C model was 0.53% v/v and errors of the same order of magnitude were obtained at other temperatures (15, 35 and 40°C). And an *r* ^2^ better than 0.99 was achieved for each validation set. The possibility and accuracy of using the miniature SW-NIR spectrometer and GLSW transfer calibration method for alcohol determination at different temperatures were proven. And the analysis procedure was simple and fast, allowing a strict control of alcohol content in the wine industry.

## 1. Introduction

The short-wave near-infrared (SW-NIR) region, 700–1100 nm, is suitable for nondestructive or noninvasive analysis of biological and biomedical materials [1–3]. Compared with long-wave NIR region (1100–2526 nm), the short-wave near-infrared can penetrate more deeply into a sample with less heating effect and in particular the interference arising from the intense water bands can be diminished [4]. In practical, the low cost of SW-NIR technology is a big advantage. The inexpensive tungsten lamps, LEDs, or the sunlight can be utilized as the SW-NIR light source and the light can be transmitted through inexpensive glass optical fibers. Moreover, the price of multichannel detectors, such as silicon charge-coupled device (CCD) and photo-diode array (PDA), is getting lower and lower with the increment of performances.

CCD detector is a solid-state device, and the geometry and stability of the pixels are ultrastable. CCD-based spectrometers have advantages in terms of size, robustness (no moving parts), and acquisition rate [5, 6]. Some commercial spectrometers are based on CCD technology (e.g., Ocean Optics and Boston Advanced Technologies, Inc.). Thus, the SW-NIR system based on CCD is cost effective for the comprehensive industry applications.

 In the wine industry, the alcohol content level needs to be determined continually to resolute the end point of fermentation. Alcohol meters are commonly used for this purpose. Although the method is simple, inexpensive and free of other chemical, the disadvantages are obvious: (1) the required distillation is time consuming; (2) a carefully calibration is needed according to temperature tables; (3) it is sample consuming. Thus, a fast, low-cost, and reliable alternative is desirable. The NIR methods developed for alcohol determination have been reported in terms of beverages [7, 8], beer [9], and fuels [10].

 However, the NIR method for alcohol determination in aqueous samples must take into account the influence of external variables such as temperature [11, 12]. Since the temperature can lead a change in the vibration spectra, a multivariable calibration model will not have good prediction ability without the temperature correction. Several kinds of calibration strategies were reported for this purpose, such as global model building [13] and simulated annealing [14]. Some spectra standardization method, such as piecewise direct standardization (PDS) [15], orthogonal signal correction (OSC) [16], dynamic orthogonal projection (DOP) [17], and generalized least squares weighting (GLSW) [18], can also be utilized. These methods provide means to mitigate the effect of interference arising from background chemical or physical species, systematic sampling errors, and instrumental drift [19].

 In this paper, a low cost and compact prototype of short-wave NIR spectrometer module has been designed for the alcohol determination during wine fermentation. A compact and robust structure was obtained by special optical and electrical design. The testing results of signal-to-noise ratio, stray light level, and baseline stability were presented. The designed system was applied for alcohol determination in combination with partial least square (PLS) calibration. Ethanol aqueous solutions were prepared in concentration range from 30% to 70% (v/v) and determined at different temperatures. GLSW method was utilized to correct the temperature effect and compared with the direct transfer calibration.

## 2. Experimental

### 2.1. Multichannel SW-NIR Spectrometer

**Figure 1:**
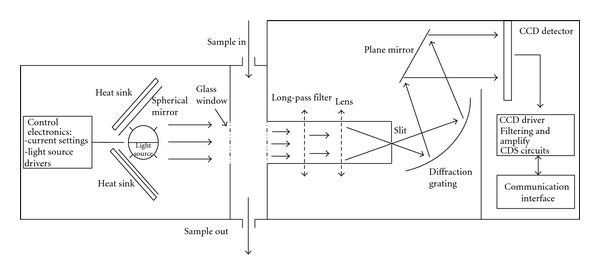
Schematic diagram of the multichannel SW-NIR spectrometer module.

The schematic diagram and the operation principle of the transmittance measurement were illustrated in Figure 1.

As shown, the light source is located at the focus point of a concave mirror. The radiation from the light source is paralleled and directed to the sample cell (10 mm optical length) to illuminate the sample. After that, the light is transmitted through a long-pass filter (cut-off wavelength of 650 nm), focused, and transferred out through an exit slit. The output light is directed to a fixed grating monochromator, positioned to give the required wavelength scale, and then reflected accurately to the CCD. 

 The LED-enhanced light source was designed by assembling a tungsten lamp with two narrow-band LEDs centered at the wavelengths of 920 nm and 1020 nm. During operation the control electronics drive the tungsten lamp and the LEDs independently to enhance the long-wavelength intensity.

 The grating polychromator optics are of a fixed construction. They contain a convex lens, entrance slit, concave grating, and a reflectance mirror. The main optical specifications are a focal length of 48 mm, a grating groove frequency of 1200 mm^−1^, a numerical aperture of 0.6, and detection array width of 24 mm. By using a Si CCD (model ILX511) [20], the maximum optical resolution is obtained of 0.24 nm with wavelengths ranging from 650 to 1100 nm. The typical integration time is 2 ms and can be regulated from 1 ms to 10 seconds.

### 2.2. Sample and SW-NIR Procedure

Samples were obtained by using aqueous solution of ethanol. 20 samples were prepared at 25°C in the concentration range of 30%–70% (v/v). They were split into two subsets, 10 pieces for calibration and 10 for validation. Each sample in the validation set was split into 4 portions and saved at 15°C, 25°C, 35°C, and 40°C, respectively. The calibration set was saved at 25°C. Every sample was collected in a glass bottle with a cap to prevent volatilization.

Transmittance NIR spectra were recorded with an optical resolution of 1 nm and at the integration time of 20 ms, accumulating 16 scans. The transmittance NIR spectrum of air at room temperature was used as the background. Total 50 spectra were recorded, 10 for calibration set at 25°C, 10 for validation set at 15°C, 25°C, 35°C, and 40°C, respectively.

 Alcohol meter (LM79-J10, Beijing, China) was used for the reference method. The temperature was kept constant by using a thermometer immersed in the samples with a precision of 0.1°C, and the samples were placed in water bath. Self-writing software was used for the spectrometer control and data collection. MATLAB7.9 (MathWorks, Inc., Natick, MA, USA) with PLS_Toolbox 6.2 from Eigenvector Research Inc. (3905 West Eaglerock Drive, Wenatchee, WA 98801, USA; http://www.eigenvector.com/) was used for data processing.

## 3. Results and Discussion

### 3.1. Characteristics of Developed Spectrometer

#### 3.1.1. Signal-to-Noise Ratio

**Figure 2:**
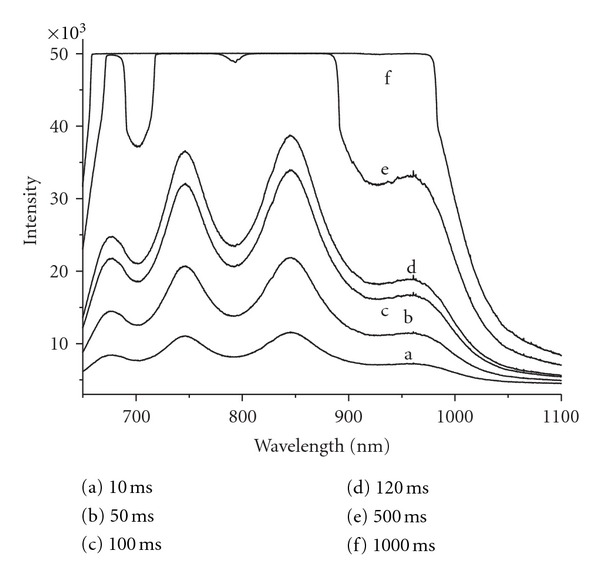
Light source spectra recorded for different integration times.

Signal-to-noise ratio is the most important parameter of an NIR spectrometer. It can be improved by enhancing the signal intensity or reducing the noise level. In this paper, the signal intensity was improved by controlling the CCD integration time and changing the light source spectrum characteristics. As shown in Figure 2, the signal intensity increased with the integration time.

The intensity in some wavelengths reached maximum with the 500 ms integration time. In practical, the appropriate integration time can be resolved when any of the CCD pixels provided the maximum signal output. And the spectrum recorded in this integration time can be used as the background. However, as shown in Figure 2, the signal intensity was not consistent in the whole wavelength range. To enhance the signal level, especially in the region after 900 nm, two LEDs were utilized. 

**Figure 3:**
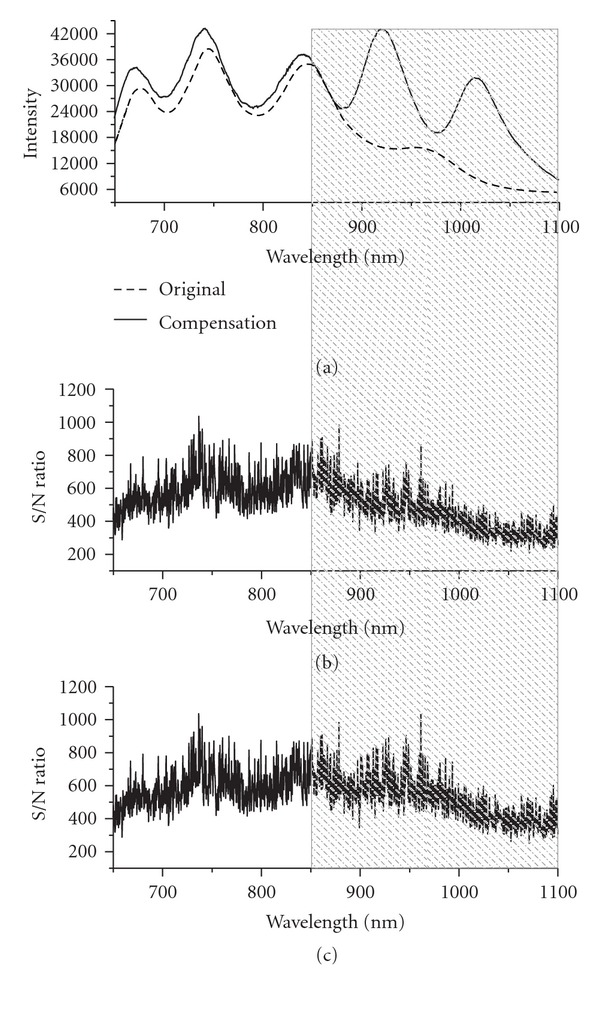
(a) Light source spectrum before and after compensation, (b) signal-to-noise ratio before compensation (c) signal-to-noise ratio after compensation.

The comparison of the light source spectra was illustrated in Figure 3(a). It can be seen that the signal was significantly enhanced in the long-wavelength region (the shadow region). The signal-to-noise ratio in the whole wavelength range was calculated and compared by recording the same spectrum for 20 times. The average intensity was calculated as the signal value and the variance as the noise level. As shown in Figure 3(b) and Figure 3(c), the signal-to-noise level increased in the shadow region. The average signal-to-noise ratio of the whole spectrum rose from 460 to 500, getting 9% increase. 

#### 3.1.2. Wavelength Accuracy and Stray Light

The spectrometer was calibrated by using lasers at 808 nm and 960 nm. Monochromatic lights centered at 718 nm, 820 nm, 902 nm, 992 nm, and 1078 nm were used to test the wavelength accuracy and stray light of the spectrometer module. The typical band widths at half maximum of the five monochromatic lights were 8 nm. As seen in Table 1, standard deviations of the tested five wavelengths were under 0.22 nm (*n* = 20).

The stray light was calculated as maximum value outside the ± 18 nm region, centered at the pass-band and presented in percent, relative to the peak of the spectrum. The stray light was found to range from 0.03% to 0.65% depending on the wavelength, as listed in Table 1. The unit-to-unit repeatability of stray light was better than ±0.1% (±2*σ*) for the tested wavelengths.

#### 3.1.3. Baseline Stability

The baseline stability of the developed spectrometer was tested by recording the baseline every five minutes during 100 minutes. The baseline was defined as the absorbance spectrum of air with itself as the background. The baseline tended to be more stable in long wavelengths. It increased from −0.07 db to 0.12 db near 700 nm, while the shifting became very tiny in the region around 1000 nm. The variance of the recorded baselines ranged from 0.0087 db at 1017.2 nm to 0.0705 db at 641.8 nm with an average value of 0.0296 db. The main specifications of the developed SW-NIR spectrometer were listed in Table 2.

### 3.2. Alcohol Determination

#### 3.2.1. Direct Transfer Calibration

**Figure 4:**
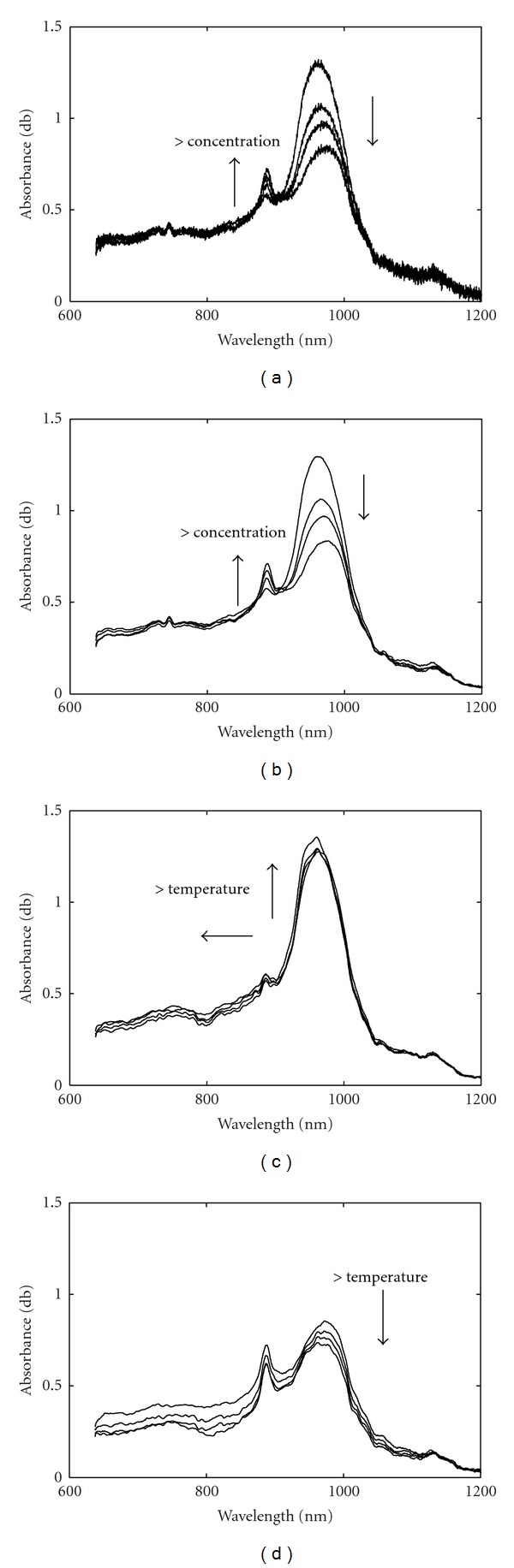
(a) Spectra of four alcohol contents at 25°C; the arrows indicate the increase of concentration. (b) the smoothed spectra of (a). (c) Spectra of 25% (v/v) alcohol at four temperatures; the arrows indicate the increase of temperature. (d) Spectra of 75% (v/v) alcohol at four temperatures; the arrow indicates the increase of temperature.

 The NIR spectra of ethanol aqueous solutions for various alcohol contents and temperatures were shown in Figure 4. The original spectra of four alcohol contents at 25°C were illustrated in Figure 4(a) and in Figure 4(b) was the smoothed spectra based on a Savitsky-Golay filter [21]. As shown, an increase in the alcohol content leads to increase of the intensity of the small band around 900 nm and decrease at 950–1000 nm absorbance.  Figure 4(c) shows the spectra of 25% alcohol content at four temperatures. As seen, the increase of temperature leads to increase of 950–1000 nm absorbance and a shift to the short wavelength.  Figure 4(d) shows the temperature effect to higher alcohol content (75% v/v) at four temperatures. As shown, an increase of temperature also leads to a baseline shift. The analysis shows that there is a dependence of the bands on alcoholic content and that a temperature correction seems to be necessary for a more general calibration model.

 PLS calibration was arranged in the wavelength range of 800–1100 nm. The spectra were smoothed and mean centered prior to modeling. Models at 25°C were cross-validated by leave-one-out strategy, tested by using the 25°C validation set, and then used to predict the validation set at other temperatures. The latent variable (LV) number was selected according to root mean square error of cross-validation (RMSECV) and the optimized value was 2. As shown in Table 3, the two latent variables were selected, because more LVs did not produce significant differences in RMSECV but lead to a higher RMSEP. 

**Figure 5:**
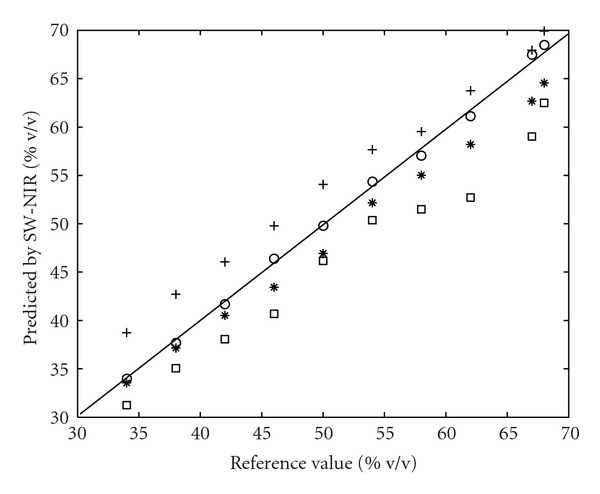
Reference value versus SW-NIR-predicted value by using 25°C model. Circle: validation set at 25°C; Plus: validation set at 15°C; Star: validation set at 35°C; Square: validation set at 40°C.

**Figure 6:**
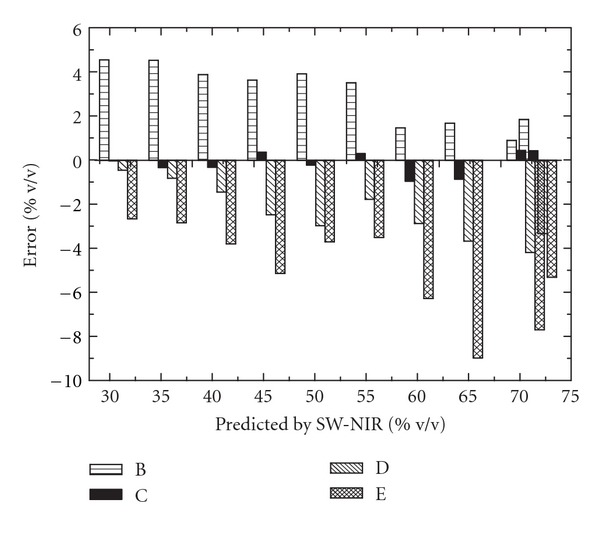
The prediction error for various temperature validations. B: validation set at 15°C; C: validation set at 25°C; D: validation set at 35°C; E: validation set at 40°C.

The prediction results with a reference method were shown in Figure 5. The coefficient of determination (*r* ^2^) for 25°C was 0.99, which showed a quite satisfactory correlation. However, as shown, results were poor for the same validation set at other temperatures. It was possible to see in Figure 6 the presence of systematic errors due to changes of temperature (B, C, D, and E denote temperature of 15, 25, 35, and 40°C, resp.). As shown, a positive systematic error was found when the validation set was at the lower temperature than the calibration set, while a negative systematic error was found when the validation set temperature was higher.

#### 3.2.2. Temperature Correction

 A transfer calibration method, GLSW, was applied to arrange the temperature correction. Calculating a filter matrix based on the differences between groups of samples which should otherwise be similar, GLSW helps to downweight the differences and make the spectra of the same sample at different temperatures appear more similar. The GLSW procedure was performed by building a GLSW model using selected samples in every temperature. Five samples were selected in each validation set according to the Euclidean distance to build the GLSW model. After that, all the samples were filtered by the GLSW model, and a model was rebuilt using the filtered 25°C calibration set. The parameter, *α*, defining how strongly GLSW downweighs interferences, was adjusted. Larger values decrease the effect of the filter, while smaller *α* applies more filtering. As shown in Table 4, 0.01 was the best value in this case, providing RMSEP of 0.74, 0.55, 0.82, and 0.89 (% v/v) for 15, 25, 35, and 40°C, respectively.

**Figure 7:**
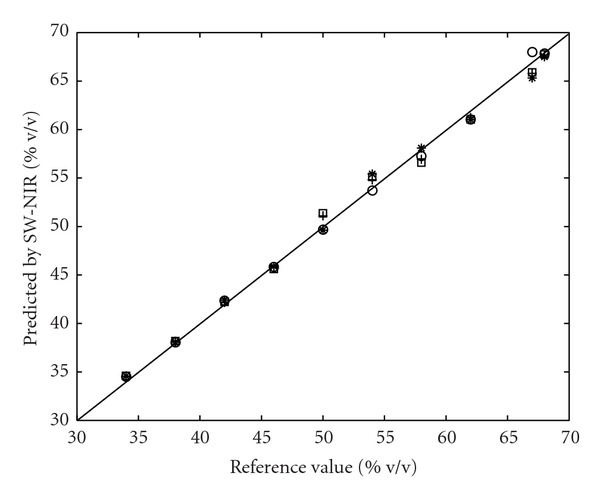
Reference value versus SW-NIR predicted value by using GLSW filter. Circle: validation set at 25°C; Plus: validation set at 15°C; Star: validation set at 35°C; Square: validation set at 40°C.

As shown in Figure 7, after the GLSW filter, good corrections for all the four temperatures were obtained, *r* ^2^ for 15, 25, 35, and 40°C was 0.997, 0.997, 0.996, and 0.996, respectively.

**Figure 8:**
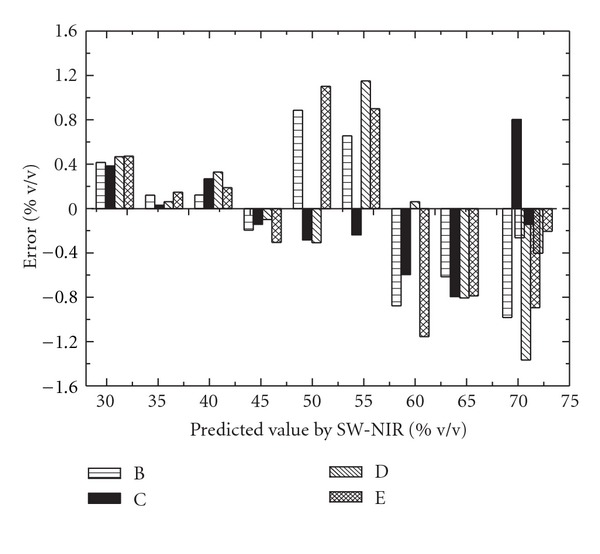
The prediction error for validation sets at various temperatures by using GLSW filter. B: validation set at 15°C; C: validation set at 25°C; D: validation set at 35°C; E: validation set at 40°C.

In terms of RMSEP for 15, 25, 35 and 40°C validation sets, 3.39%, 0.52%, 2.77% and 5.57% were obtained for no-temperature corrected model, respectively. While, for the model using GLSW filter, far more superior results were obtained, 0.74%, 0.55%, 0.82%, and 0.89%, respectively, as shown in Table 4. And with this correction, the previously appeared systematic errors disappeared, as shown in Figure 8, indicating the use of GLSW in this case, a good method to correct for eventual problems related to temperature effect.

## 4. Conclusion

A multichannel short-wave NIR spectrometer was developed based on CCD detection and tested for performance evaluation. By optimizing the light source design, the signal-to-noise ratio, which is the most important parameter of an NIR spectrometer, kept higher values in the whole wavelength range. The possibility and accuracy for alcohol determination in wine industry by using this inexpensive spectrometer were verified. To avoid errors raised by different temperatures, a correction method is necessary. And the GLSW was proved to be a satisfactory method with a view to decreasing errors in relation to direct transfer calibration.

## Figures and Tables

**Table 1:** Statistic result of the five tested wavelengths.

Nominal (nm)	Measured (nm)*	Residuals (nm)	Standard deviation (nm)	Straylight (%)
718 820 902 992 1078	716.80 820.83 902.48 992.24 1079.20	−1.20 0.83 0.48 0.24 1.20	0.19 0.17 0.06 0.20 0.22	0.22 0.03 0.04 0.12 0.65

*All the values were the averages of 20 times results.

**Table 2:** The specifications of the developed SW-NIR spectrometer.

Items	Specifications
Wavelength range	650–1100 nm
Signal-to-noise ratio	500 (full wavelength)
Maximum optical resolution	0.24 nm
Wavelength accuracy	<1.2 nm
Wavelength precision	<0.22 nm
Optical stray light	<0.65%
Repeatability of straylight	<0.1%
Integration time	1 ms–10 sec (typical 2 ms)
Baseline stability	0.0296 db (average)

**Table 3:** Results for calibration model at 25°C.

LV	RMSEC %	RMSECV %	RMSEP %
1	1.28	1.69	1.30
2	0.34	0.36	0.52
3	0.13	0.28	0.67
4	0.06	0.27	0.66

**Table 4:** Results of GLSW model for different *α* values.

*α*	RMSEP % 15°C	RMSEP % 25°C	RMSEP % 35°C	RMSEP % 40°C
0.001	0.81	0.55	0.81	0.95
0.01	0.74	0.55	0.82	0.89
0.1	0.80	0.60	1.23	1.20

## References

[1] Ozaki Y, Matsunaga T, Miura T (1992). Nondestructive and noninvasive monitoring of deoxyhemoglobin in the vein by use of a near-infrared reflectance spectrometer with a fiber-optic probe. *Applied Spectroscopy*.

[2] Šašić S, Ozaki Y (2001). Short-wave near-infrared spectroscopy of biological fluids. 1. Quantitative analysis of fat, protein, and lactose in raw milk by partial least-squares regression and band assignment. *Analytical Chemistry*.

[3] Sato H, Wada S, Ling M, Tashiro H (2000). Noninvasive measurement of oxygenation of hemoglobin by direct transmission of near-infrared energy (700-1000 nm) from an electronically tuned Ti:sapphire laser driven by a dual radio-frequency driving method. *Applied Spectroscopy*.

[4] Reeves JBJ (1994). Effects of Water on the Spectra of Model. Compounds in the short-wavelength near-infrared spectral region. *Journal of Near Infrared Spectroscopy*.

[5] Alves J, Santos JL, Carvalho A, Lage A Fiber bragg sensor interrogation system based on a CCD spectrometer.

[6] Deckert V, Kiefer W (1992). Scanning multichannel technique for improved spectrochemical measurements with a CCD camera and its application to raman spectroscopy. *Applied Spectroscopy*.

[7] Nagarajan R, Gupta A, Mehrotra R, Bajaj M (2006). Quantitative analysis of alcohol, sugar, and tartaric acid in alcoholic beverages using attenuated total reflectance spectroscopy. *Journal of Automated Methods and Management in Chemistry*.

[8] Barboza FD, Poppi RJ (2003). Determination of alcohol content in beverages using short-wave near-infrared spectroscopy and temperature correction by transfer calibration procedures. *Analytical and Bioanalytical Chemistry*.

[9] Castritius S, Kron A, Schäfer T, Rädle M, Harms D (2010). Determination of alcohol and extract concentration in beer samples using a combined method of near-infrared (NIR) spectroscopy and refractometry. *Journal of Agricultural and Food Chemistry*.

[10] Dorado MP, Pinzi S, de Haro A, Font R, Garcia-Olmo J (2011). Visible and NIR Spectroscopy to assess biodiesel quality: determination of alcohol and glycerol traces. *Fuel*.

[11] Wülfert F, Kok WT, Smilde AK (1998). Influence of Temperature on Vibrational Spectra and Consequences for the Predictive Ability of Multivariate Models. *Analytical Chemistry*.

[12] Barboza FD, Poppi RJ (2003). Determination of alcohol content in beverages using short-wave near-infrared spectroscopy and temperature correction by transfer calibration procedures. *Analytical and Bioanalytical Chemistry*.

[13] Wulfert F, Kok WT, De Noord OE, Smilde AK (2000). Correction of temperature-induced spectral variation by continuous piecewise direct standardization. *Analytical Chemistry*.

[14] Swierenga H, Wülfert F, De Noord OE, De Weijer AP, Smilde AK, Buydens LMC (2000). Development of robust calibration models in near infra-red spectrometric applications. *Analytica Chimica Acta*.

[15] Bouveresse E, Massart DL (1996). Standardisation of near-infrared spectrometric instruments: a review. *Vibrational Spectroscopy*.

[16] Chen D, Hu B, Shao X, Su Q (2004). Removal of major interference sources in aqueous near-infrared spectroscopy techniques. *Analytical and Bioanalytical Chemistry*.

[17] Zeaiter M, Roger JM, Bellon-Maurel V (2006). Dynamic orthogonal projection. A new method to maintain the on-line robustness of multivariate calibrations. Application to NIR-based monitoring of wine fermentations. *Chemometrics and Intelligent Laboratory Systems*.

[18] Martens H, Høy M, Wise BM, Bro R, Brockhoff PB (2003). Pre-whitening of data by covariance-weighted pre-processing. *Journal of Chemometrics*.

[19] Wise BM, Shaver JM, Gallagher NB Using clutter to improve pattern recognition, calibration and classification models.

[20] SONY 2048-pixels CCD linear image sensor (B/W) ILX511. Japan.

[21] Savitzky A, Golay MJE (1964). Smoothing and differentiation of data by simplified least squares procedures. *Analytical Chemistry*.

